# The intoxicated co-witness: effects of alcohol and dyadic discussion on memory conformity and event recall

**DOI:** 10.1007/s00213-021-05776-0

**Published:** 2021-02-10

**Authors:** Georgina Bartlett, Julie Gawrylowicz, Daniel Frings, Ian P. Albery

**Affiliations:** 1 Centre for Addictive Behaviours Research, Division of Psychology, London South Bank University, 103, Borough Road, London, SE1 0AA UK; 2 Division of Psychology and Forensic Science, Abertay University, Dundee, UK

**Keywords:** Alcohol, Co-witness discussion, Memory conformity, Misinformation, Intoxication

## Abstract

**Rationale:**

Co-witness discussion is common and often witnesses are under the influence of alcohol. As such, it is important to understand how such factors may influence eyewitness testimony.

**Objectives:**

We combined a co-witness memory paradigm with an alcohol administration paradigm to examine the influence of alcohol and dyadic discussion on remembering a mock crime.

**Methods:**

Intoxicated and sober dyads discussed a previously seen video, whilst in a control condition sober and intoxicated individuals recalled the event on their own. Unknown to the dyads, each discussion partner saw a different version of the video including unique details not present in the other video version. All participants then engaged in a second individual recall attempt.

**Results:**

Dyads were more likely to recall misleading details in their individual recall attempts compared to the control group. Intoxicated and sober dyads were equally likely to report misleading information. Alcohol intoxication had no negative impact on individuals’ ability to correctly identify the source of their responses. Intoxicated participants recalled fewer details under free recall conditions. Alcohol had a detrimental effect on participants’ confidence in their free recall accounts.

**Conclusions:**

Possible alcohol-related and social-cognitive mechanisms are discussed which may contribute to the current findings as well as applied implications for interviewing intoxicated witnesses.

Instances where multiple witnesses and victims are present are common. Skagerberg and Wright (2008) reported that 88% of witnesses surveyed had at least one ‘co-witness’ to the crime and of those, 58% reported discussing details of the event with other witnesses. Other work shows that 60% of violent crimes occur at weekends around pubs/clubs (Allen et al. 2003), suggesting that victims and witnesses might be under the influence of alcohol. Also, in about 44% of UK police interviews conducted per month, the victim was drunk at the time of the crime (Crossland et al. 2018). Together, this research suggests that discussion amongst intoxicated witnesses to a crime prior to having their statement taken by police may not be uncommon and demonstrates the need to investigate the combined effect of intoxication and discussion on eyewitness memory accounts.

Memory distortions that are the result of misleading post-event information (PEI) acquired through discussion with a partner are referred to as *memory conformity* (Gabbert et al. 2003). For example, Gabbert et al. (2003) found that after viewing a video of a mock crime, 71% of dyads who had been exposed to misinformation during a discussion with a partner went on to recall at least one item that they did not see but only heard about from their co-witness. One’s susceptibility to memory conformity is influenced by numerous factors including the age of the discussion partner (Meade et al. 2016), personality traits (Doughty et al. 2017), familiarity with and trust in the discussion partner (Condon et al. 2015), negative feedback (Monds et al. 2019), co-witness confidence and one’s own confidence (Thorley and Kumar 2016). One influencing factor that has been somewhat overlooked in the memory conformity literature is the effect of alcohol intoxication.

Research examining the effects of alcohol and suggestibility has revealed mixed findings. For instance, Schreiber Compo et al. (2012) showed that sober and intoxicated participants (BAC = 0.07%) were equally susceptible to reporting misinformation when recalling memories about a live staged theft after overhearing a telephone conversation which introduced misinformation. That intoxication (BAC = 0.06%) does not increase the propensity to recall misinformation was also shown by Flowe et al. (2019) using a hypothetical sexual assault paradigm. Similarly, Thorley and Christiansen (2018) found that intoxicated participants (mean BAC = 0.06%) were no more prone to reporting erroneous suggestions from a sober confederate than sober participants. In contrast, laboratory research by Evans et al. (2019) found that participants who were intoxicated at encoding were significantly more likely to agree with incorrect suggestions when recalling after a 1-week delay than their sober counterparts. Moreover, fieldwork with severely intoxicated participants (mean BAC = 0.16%) showed that they were more susceptible to misleading questions compared to sober participants (Van Oorsouw et al. 2015; Van Oorsouw et al. 2019). Together, this work shows that, using narratives (Flowe et al. 2019), confederates (Thorley and Christiansen 2018) and misleading questions (Van Oorsouw et al. 2015, 2019) as sources of misleading PEI, at moderate intoxication levels, individuals appear to be no more likely to accept misinformation, but at higher doses misinformation may be incorporated into their memory reports.

The effect of alcohol on memory conformity during a face-to-face interaction, where both dyad partners are intoxicated or sober, has not been explored. The current research combined a memory conformity paradigm with an alcohol administration paradigm. In the typical memory conformity paradigm (Gabbert et al. 2003), participants watch one of two versions of a mock crime, but they are made to believe that they watch the same video. Importantly, unknown to the participants, the videos differ slightly with regard to what kind of details can be seen. For instance, an opportunistic theft taking place in one video that cannot be seen in the other (Gabbert et al. 2003). Participant dyads then discuss what they remember, thereby potentially exposing each other to misleading PEI. After the ‘social’ encounter, participants engage in an individual recall session to examine whether they incorporated the PEI into their own memory accounts (Gabbert et al. 2003, 2004). A ‘no discussion’ individual control condition is also included to compare the effects of co-witness discussion versus no discussion on subsequent memory reports (Gabbert et al. 2003; Hope et al. 2008; Paterson and Kemp 2006).

In line with previous memory conformity studies (French et al. 2008; Gabbert et al. 2003), we hypothesised that participants who discussed the event in pairs would incorporate more PEI in their subsequent individual reports than participants who had recalled the event alone. In addition, we investigated whether intoxicated pairs were more susceptible to memory conformity than sober ones. Given that some studies have found that intoxicated mock witnesses are more susceptible to misleading suggestions (Van Oorsouw et al. 2015, 2019), we predicted that intoxicated dyads would be more prone to reporting PEI. However, Thorley and Christiansen (2018) showed that intoxicated co-witnesses may be perceived as less credible by their discussion partner and participants might therefore be less likely to accept PEI from them. Hence, an alternative hypothesis was that intoxicated dyads would be less susceptible to reporting PEI compared to sober ones. Finally, we also examined the completeness and accuracy of participants’ accounts. In line with a recent meta-analysis by Jores et al. (2019), we expected that a moderate dose of alcohol, as used in the current study, leads to less complete but not less accurate accounts. Finally, alcohol intoxication may impact an individual’s metacognition (Gawrylowicz et al. 2018). We therefore explored how source monitoring abilities and confidence judgments were influenced by alcohol and dyadic discussion.

## Method

### Participants and design

One hundred twenty-two participants (106 females and 16 males, mean age 24.10 years, SD = 7.67) took part in the study in exchange for course credit. We used a 2 (discussion: dyad vs. individual condition) × 2 (beverage: intoxicated vs. sober condition) between-subject design. The dependent variables were susceptibility to memory conformity, completeness and accuracy of the free and cued recall, source monitoring and confidence judgments. The study received ethical approval from the School of Applied Sciences’ Ethics Committee at X (location redacted for purposes of blind review). An achieved power analysis computed using G* Power version 3.1.9.6. (Faul et al. 2007), indicated that to detect main effects and interactions in a 2 × 2 between-subject design, a sample of 122 participants had a power of 0.78, with a critical *F* of 3.92.

### Materials

#### Screening questionnaire

Participants completed the Alcohol Use Disorders Identification Test (AUDIT) (Saunders et al. 1993). The AUDIT is a ten-item questionnaire measuring harmful alcohol consumption. It requires participants to consider how often they have engaged in certain drinking practices over the past 12 months. For example, participants were asked whether they have consumed more than six drinks on one occasion or found that they could not stop drinking once they had started. Scores range from 0–40, with a score of 8+ suggesting harmful drinking behaviour. Participants were also asked if they had an existing medical condition, were taking any medication, or if there was a chance that they could be pregnant. One participant was excluded from taking part because of taking medication that could potentially interfere with alcohol intake.

#### Drinks and breath alcohol measurement

Participants in the intoxicated condition were given cups of vodka and orange juice in a 2:1 ratio. This dose was calculated using participants’ gender and bodyweight as 0.6g/kg in order to achieve a target BAC of 0.06% with a maximum dose of 175 ml of 37.5% abv vodka. Participants in the sober condition were given orange juice equivalent to the total volume of the alcohol beverage to drink. Blood alcohol levels were assessed with a Lion Alcometer 500 breathalyser.

#### Videos

Stimuli were derived from Gabbert et al. (2003). Both videos depicted a student entering an academic office to drop off a book. She looks for a pen to leave a note whereupon she finds a wallet with money inside of it. Both videos depicted the same event but were shot from different viewpoints with slightly different details visible. Video A included two critical items that were not visible in video B. The student scrunching a note up and throwing it in the bin and the title of the student’s book, ‘memory disorders’, was visible. Video B also included two critical items that were not visible in video A. The student is looking at her watch and also carrying out an opportunistic crime (stealing money from the wallet). In total, there were four pieces of misinformation (as in Gabbert et al. 2003), two in each video.

#### Recall questionnaire

A questionnaire was used to structure the discussion and individual recall phase. It consisted of a free and cued recall section (see Gabbert et al. 2003). For the free recall, participants were asked to think back to the video and imagine they had witnessed the event in real life and need to recall all of the details they can remember in order to provide information to the police. After completing the recall, participants were required to indicate their confidence in the statement on a 1–7 Likert scale. The cued recall section consisted of questions pertaining to ‘neutral’ items (i.e. items that were visible in both video versions, for example the colour of her bag) as well as ‘critical’ items (i.e. items seen in one video but not the other, for example the title of the book). Participants were asked to indicate their confidence in their responses after each question. A single source monitoring question at the end of the recall questionnaire required participants in the dyad condition to indicate whether their answers were based upon the video, the discussion partner or both. Source monitoring responses were scored as incorrect when participants reported misinformation but stated that they only included answers based upon the video. Responses were scored as correct when participants reported misinformation and stated that they based their answers on their discussion partner or both their discussion partner and the video. Responses were also scored as correct if participants did not report any misinformation and stated that they only based their responses upon the video.

### Procedure

#### Alcohol administration phase

Prior to the study, all participants were advised to not eat for 3 h. Participants arrived at the lab and were provided with a study information sheet and completed the screening to ensure eligibility for participation. Consent forms were signed, and participants were informed that they would be required to drink either an alcoholic or non-alcoholic beverage and weighed (for calculating dosage). Participants in the sober condition were told that their drink contained orange juice and were given orange juice. Participants in the intoxicated condition were told that their drink contained vodka and were given a vodka orange juice mixer. All participants were breathalysed prior to the consumption phase to ensure that they were completely sober. Participants were informed that they had to consume their drinks within 30 min but not faster than 20 min (see Gawrylowicz et al. 2017, 2018). Participants in the dyad condition consumed the beverage together with their discussion partner. After alcohol consumption, participants were given a small amount of water to rinse their mouths to remove any residual alcohol that could contaminate breathalyser readings. After 10 min, participants were then breathalysed, at which point the study commenced if their BAC had reached the desired target level (0.06%) or were breathalysed again after 5 min (an average of the two readings was then used for all further calculations).

#### Video and recall phase

##### Dyad condition

Participants in the dyad condition were invited one at a time to watch a short video. Participants were informed that they would be watching the same video but that they must watch it one at a time, as there was only one monitor. The second participant sat on the other side of the laboratory desk whilst waiting for their turn to watch the video. Participants were then instructed to work together to generate the most accurate and complete account of the events seen. The recall questionnaire was provided to aid the discussion. Participants were informed that they had 10 min to complete the questionnaire together. After 10 min had passed, any dyads that had not yet finished were encouraged to finish up their discussion. They were then given a filler task to complete for 15 min, after which they were breathalysed again prior to completing their individual recall. All participants then completed the recall questionnaire again individually. Finally, participants were fully debriefed. Participants in the intoxicated condition were breathalysed again, and those with BrACs of > 0.1mg/l were advised to stay in the laboratory until their BrAC reduced to below 0.1 mg/l. Those who wished to leave were asked to sign an exit waiver to acknowledge that they were aware of the risks in doing so.

##### Individual condition

In the individual condition, participants were randomly assigned to watch either video A or video B alone. They then recalled the details of the video using the recall questionnaire individually rather than engaging in a discussion. Subsequently, the experimental procedure was the same for individuals and dyads. All participants (individuals and dyads) completed the filler task for 15 min before completing the individual recall questionnaire. The duration of the experiment was the same for individuals and dyads, with equivalent timings used for individual recall vs discussion. As such, no differences in BAC or retention interval would be expected between participants in the individual and dyad condition.

### Data scoring

A coding sheet was produced listing the actions and events of the video as well as details describing the environment and the surroundings seen in the video. A total of 63 details were included. The numbers of correct, errant and misinformation details were recorded for the free recall and cued recall tests separately. A detail was only scored once regardless of whether participants mentioned it multiple times. An item was classed as misinformation if it was not present in the video the participant had seen but was present in the other video version (e.g. if participants who watched video A described that the female was stealing the money although this was not depicted in video A). A detail was scored as correct if it featured in the video version seen and was accurately described (e.g. the female carried a bag and the bag was visible in the version of the video the participant saw). A detail was scored as an error when it featured in the video version seen but was incorrectly described (e.g. the female wore a red top but in fact the top was grey) or when the detail did not feature in any of the video versions seen (e.g. an accomplice was present when in fact no accomplice was present in any video versions). Units of information that did not meet the criteria for classification as either ‘accurate’, ‘error’ or ‘misinformation’ were not scored.

One researcher coded all of the scripts, however, to ensure that the coding procedure was clear and transparent, a subset of 25% were double coded for accuracy, error and misinformation details by a second coder. The second coder was trained by the researcher in the coding procedure and provided with all coding materials to code 25% of the scripts on their own. The intraclass correlation coefficient for accuracy was .98 *F*(30, 30) = 47.39, *p* < .001 95% CI [.96, .99]. For errors, the ICC was .90 *F*(30, 30) = 9.71, *p* < .001 95% CI [ .79, .95]. For misinformation, the ICC was .85 *F*(30, 30) = 6.77, *p* < .001, 95% CI [.69, .93] demonstrating excellent reliability. Disagreements between coders were resolved on a consensus basis.

## Results

### Manipulation checks

#### Alcohol intoxication

Participants BrACs were converted to BACs using a blood:breath ratio of 2300:1. Mean BACs were calculated using the post-consumption readings. For participants who required two readings, because they did not reach target levels at the first reading, an average of these two was calculated and used to compute the group average. The average BAC score amongst intoxicated participants was 0.06% (BACs ranged from 0.01 to 0.1%). All control participants had a BAC level of .00%. A *t*-test indicated a significant difference in BAC between conditions, *t*(60) = 22.1, *p <* .001. There was no significant difference in BACs between intoxicated participants assigned to the dyad vs individual condition, *t*(49.5) = 1.67, *p* = .101. The average difference in post-consumption BACs between intoxicated dyads in the dyad condition was 0.02% (range 0.00–0.05%).

#### Co-witness influence

In total, 86.7% of participants in the dyad condition received at least one piece of misinformation from their partner. To check that the memory conformity manipulation was successful, a chi-square analysis was run which demonstrated a significant association between discussion condition and recalling misinformation, *χ*^2^ (1, 122) = 25.70, *p* < .001. Participants in the dyad condition were more likely to report at least one piece of misinformation in their accounts compared to those who recalled the event individually. The memory conformity effect was found for both sober participants, *χ*^2^ (1, 61) = 15.78, *p* < .001, and intoxicated participants, *χ*^2^ (1, 61) = 10.35, *p* = .001. Also, participants were just as likely to report misinformation if they saw video A as video B, *χ*^2^ (1,122) = 1.613, *p =* .204.

#### Alcohol and memory conformity

To address the question of whether alcohol intoxication is associated with reporting misinformation, a log-linear analysis including beverage (intoxicated vs. sober), discussion (dyad vs. individual) and misinformation reported (yes vs. no) was applied to the free recall data and the cued recall data separately.

**Fig 1:**
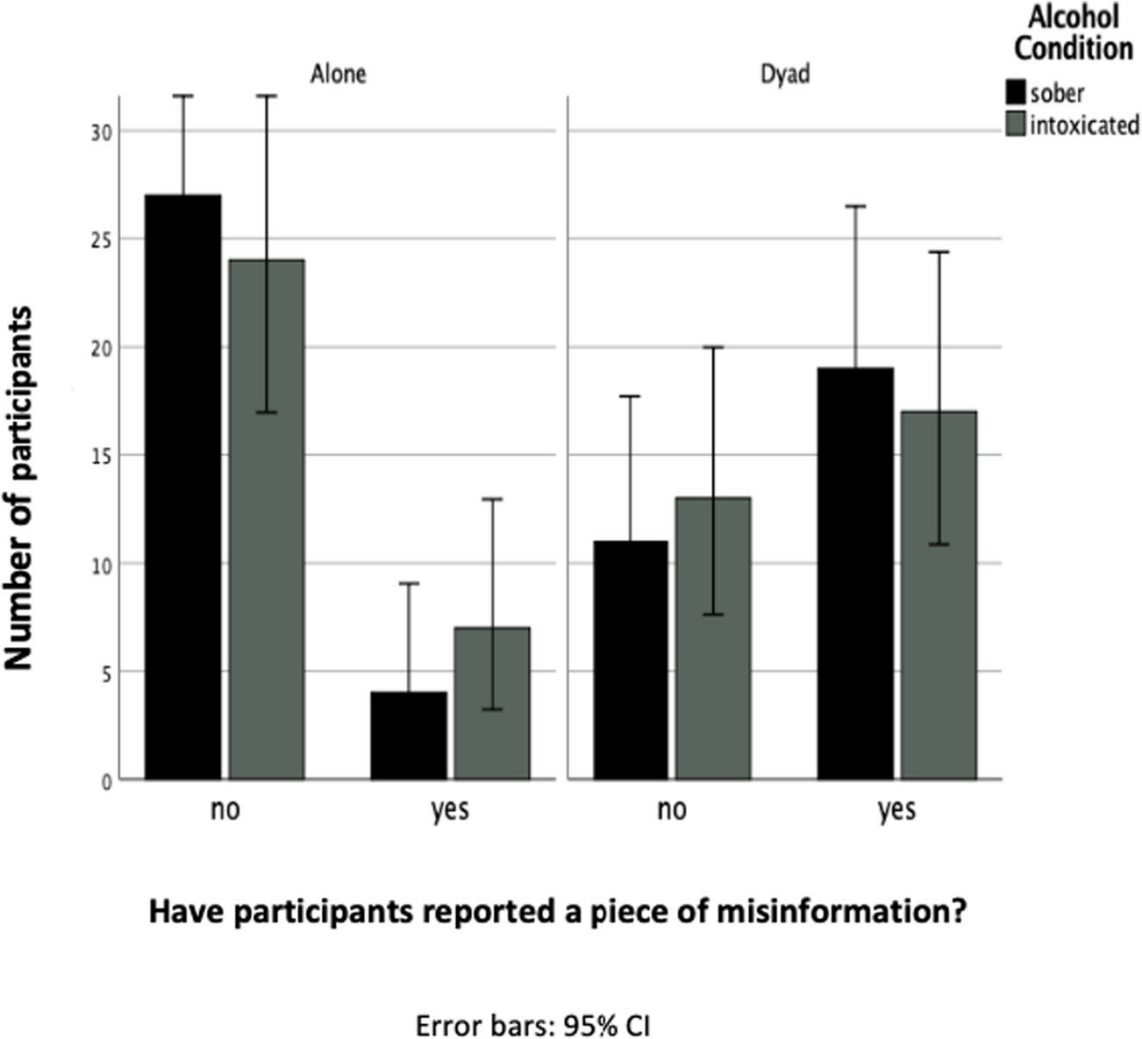
Number of participants who did and did not report misinformation in the free recall test

For the free recall data, the three-way analysis produced a final model that retained the discussion × misinformation interaction. The likelihood ratio of the model was *χ*^2^ (4) = 1.28, *p* = .864. The discussion × misinformation interaction was significant, *χ*^2^ (1) = 23.92, *p <* .001. Participants in the dyad condition were 6.95 times more likely to report misinformation than participants in the individual condition. In total, 60% of participants who had discussed the video reported misinformation. At least one piece of misinformation was reported spontaneously by 17.7% of those in the individual condition, despite not being exposed to any PEI by their discussion partner. Intoxicated dyads were no more likely to report misinformation compared to sober dyads. Figure 1 graphs the frequencies of participants including misinformation in their response. Alcohol also had no effect on the number of misinformation items reported *U* = 378.00, *p* =. 240, in the free recall.

**Fig 2:**
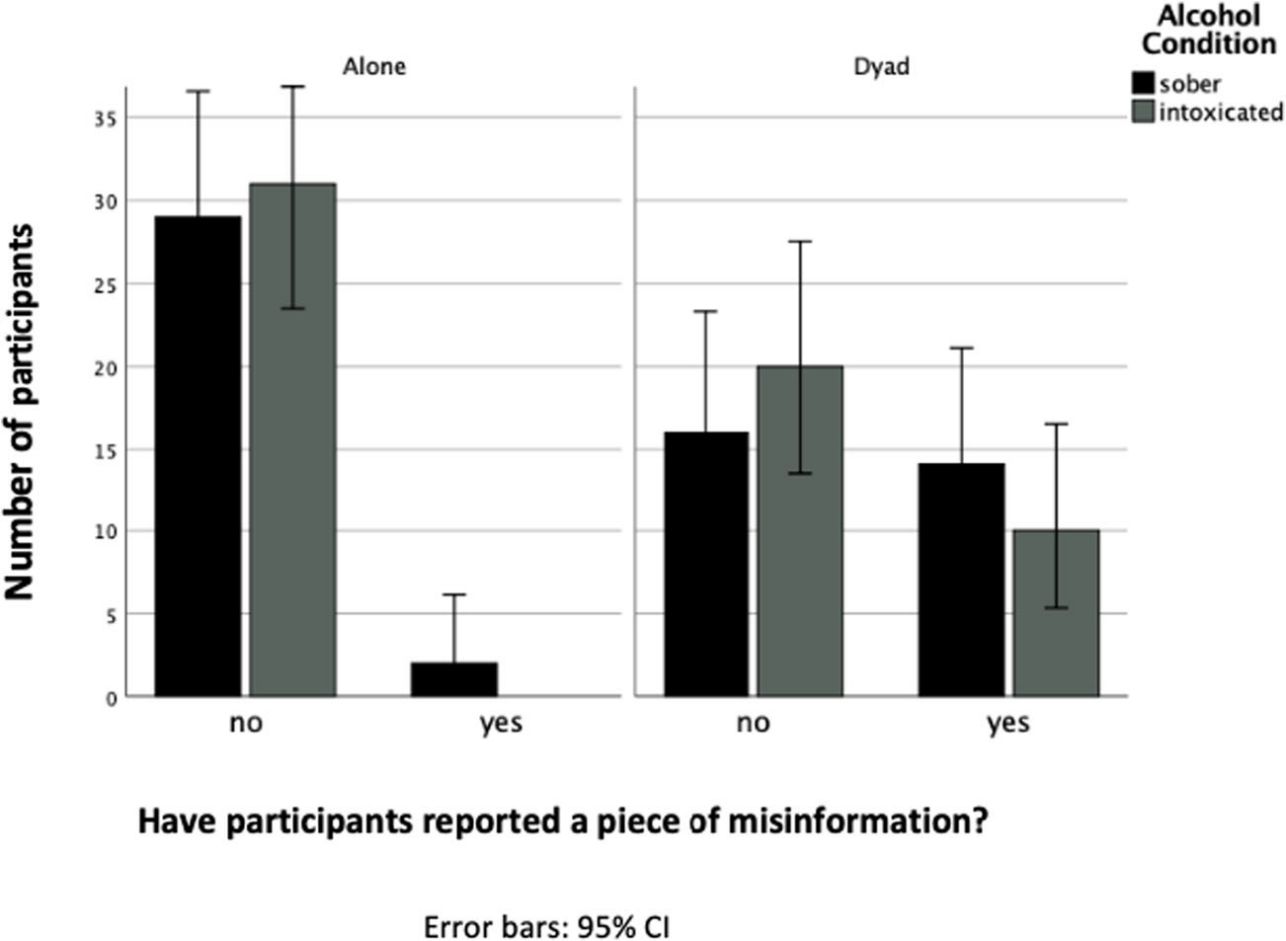
Number of participants who did and did not report misinformation in the cued recall test

Similar to the findings for the free recall data, the three-way log-linear analysis on the cued recall data produced a final model that maintained only the discussion × misinformation interaction. The likelihood ratio for this model was, *χ*^2^ (4) = 3.95, *p* = .412. The discussion × misinformation interaction was significant, *χ*^2^ (1) = 28.42, *p < .*001, demonstrating that participants who had discussed the video with a partner were more likely to include misinformation in their responses to the cued recall questions. Odds ratios indicate that participants in the dyad condition were 22 times more likely to incorporate misinformation than participants in the individual condition. In total, 40% of participants who had discussed the video reported misinformation compared to 3.20% in the individual condition. Intoxicated participants were no more likely to report misinformation than sober participants. Figure 2 shows the frequencies of participants including misinformation in their response. Additionally, as for the free recall, there was no significant difference between sober and intoxicated participants for the number of misinformation items reported to cued recall questions *U* = 380, *p* = .227.

#### Source monitoring

All participants who recalled with a partner were asked to identify whether the source for their answers was their discussion partner, the video or both. A total of 71.2% of participants were able to correctly identify the source of the information reported. Also, no association between beverage consumed and correct source monitoring, *χ*^2^ (1, 61) = 3.17, *p =.* 573, was shown*.* Intoxicated participants were as likely as sober ones to identify the source of their memory correctly.

### Memory recall

#### Completeness

To examine the effect of beverage and discussion on the completeness of participants’ free recall accounts, a 2-way ANOVA was run. There was a significant effect of beverage *F*(1, 118) = 4.48, *p = .*036 ηp^2^ = .037, with sober participants reporting more details (*M =* 23.19, SD *=* 5.97) 95% CI [21.62, 24.73] than intoxicated ones (*M =* 20.82, SD = 6.39) 95% CI [19.27, 22.38]. No significant main effect for discussion, *F*(1, 118) = 1.81, *p = .*182 ηp^2^ = .02, nor a significant beverage × discussion interaction, *F*(1, 118) = 2.20, *p= .*148 ηp^2^ = .018, were found*.* For the cued recall, there was no significant main effect of discussion *F*(1, 118) = .75, *p* = .389, ηp^2^ = .006, or beverage *F*(1, 118) = 1.97, *p* =.164, ηp^2^ =.016, nor a significant beverage × discussion interaction, *F*(1, 118) = 1.46, *p* =.230, ηp^2^ = .012.

#### Memory accuracy

Accuracy rates were computed by dividing the number of correctly reported details by the total number of details recalled. The free recall data showed no significant effects of discussion, *F*(1, 118) = 1.69, *p* =.197, ηp^2^ = .014, beverage, *F*(1, 118) = .38, *p* = .537, ηp^2^ = .00, nor a discussion × beverage interaction, *F*(1, 118) = 2.71, *p* = .102, ηp^2^ = .022, on accuracy rates. For the cued recall data, there was a significant main effect of discussion on accuracy rate, *F*(1, 118) = 4.21, *p* = .044, ηp^2^ = .034, with individual participants showing higher accuracy rates (*M* = .72, SD = .16) 95% CI [.68, .76] than those in the dyad condition (*M* =.66, SD =.19) 95% CI [.62, .70]*.* There was no significant main effect of beverage, *F*(1, 118) = .10, *p =.*756, ηp^2^ = .001, nor a significant discussion × beverage interaction, *F*(1, 118) = .37, *p* = .546, ηp^2^ = .003.

#### Confidence judgments

Participants were asked to indicate their confidence in their free recall accounts. A 2-way ANOVA revealed a significant main effect of beverage on confidence judgements, *F*(1, 114) = 9.18, *p* = .003, ηp^2^ = .075. Participants who were sober reported significantly higher confidence (*M* = 5.44, SD = 1.0) 95% CI [5.16, 5.72]) than those who were intoxicated (*M =* 4.82, SD = 1.67, 95% CI [4.54, 5.11])*.* The main effect of discussion was not significant, *F*(1, 114) = 1.04, *p* = .310, ηp^2^ = .009, nor was the discussion × beverage interaction, *F*(1, 114) = 1.79, *p = .*184, ηp^2^ = .015. Despite being no less accurate than sober participants, intoxicated participants were less confident in their accounts. For the cued recall, participants were asked to report their confidence in their answer to each question*.* Mean confidence was calculated for correct, incorrect, misinformation and ‘I don’t know’ responses. A 4 (response type: correct, incorrect, misinformation, I don’t know) × 2 (beverage: intoxicated vs sober) × 2 (discussion: individual vs discussion) ANOVA was conducted to examine the effect of response type on confidence levels. Mauchly’s test indicated that the sphericity assumption was violated, *χ*^2^ (5), 14.75, *p = .*012; therefore, the Greenhous-Geisser correction was used.

A significant main effect of response type, *F*(2.087, 29.215) = 3.42, *p* = .044, ηp^2^ = .196 was found. Bonferroni pairwise comparisons indicated that participants were significantly more confident in their correct responses than misinformation responses (*p* = .004) and ‘I don’t know’ responses (*p* = .002)*.* None of the main effects of discussion, *F*(1, 14) = 2.84, *p* =.114, ηp^2^ = .169 or beverage, *F*(1, 14) =.13, *p* = .723, ηp^2^ = .009, nor the interactions between response type and discussion, *F*(3, 29.215) = .55, *p = .*58*9*, ηp^2^ = .038, and response type and beverage, *F*(3, 29.215) = 1.33, *p = .*27*8*, ηp^2^ = .087, was significant. Mean confidence ratings for response type can be seen in Table 1.

**Table 1:** Mean confidence ratings and SDs on 1–7 Likert scale by response type, discussion condition, and beverage condition. There is one empty cell in the table as there are no cases for that cell

		Correct responses	Incorrect responses	Misled responses	I don’t know responses
Alone	Sober	5.71 (.83)	4.95 (1.30)	5.00 (1.41)	3.00 (2.22)
	Intoxicated	4.54 (1.29)	4.14 (1.71)		2.09 (2.04)
	Total	5.12 (1.23)	4.53 (1.57)	5.00 (1.41)	2.52 (2.15)
Dyad	Sober	5.02 (1.07)	4.22 (1.52)	4.65 (1.34)	2.75 (2.11)
	Intoxicated	4.94 (1.13)	4.30 (1.35)	3.60 (1.65)	2.71 (2.73)
	Total	4.98 (1.09)	4.26 (1.42)	4.20 (1.54)	2.73 (2.34)
Total	Intoxicated	4.73 (1.22)	4.22 (1.53)	3.60 (1.65)	2.34 (2.33)
	Sober	5.37 (1.01)	4.59 (1.45)	4.70 (1.31)	2.87 (2.14)

## Discussion

The current study investigated the effect of alcohol intoxication and witness discussion on memory conformity. Participants who engaged in dyadic discussion incorporated misinformation into their accounts 6.95 times more often than those who recalled alone. This effect was not influenced by alcohol intoxication. These findings highlight how susceptible individuals are to incorporate post-event information, encountered through witness discussion, into their own memory accounts. Importantly, intoxicated individuals were as susceptible to memory conformity as sober individuals. Intriguingly, a small percentage of those who recalled alone also included misinformation items, despite not being exposed to them. It could be argued that the nature of our mock-crime paradigm, including being asked to provide information to the police, has increased the likelihood of reporting that a crime happened. This would explain why a minority of participants in the individual condition later reported having seen the opportunistic theft, although this was not depicted in their version of the video.

The finding that alcohol does not increase the susceptibility to misinformation is in line with previous research (Schreiber Compo et al. 2012; Flowe et al. 2019). We extend this finding by demonstrating that this effect still applies when misinformation is presented via a dyadic discussion where both partners are intoxicated. Schreiber Compo et al. (2012) previously demonstrated that alcohol does not increase suggestibility when misinformation was presented via a phone call by the experimenter, whilst Flowe et al. (2019) showed no effect of alcohol when misinformation was presented via a written narrative. Thus, during a dyadic discussion, the tendency to report misinformation when intoxicated was the same as that shown in studies which have introduced misinformation using misleading questions or written narratives (Schreiber Compo et al. 2012; Van Oorsouw et al. 2015). It could be argued that at low to moderate intoxication levels, the source of misinformation has no or only little effect on an individual’s likelihood to incorporate misinformation in their subsequent memory reports.

In line with other work, the present study, also found that consumption of alcohol was not associated with a decrease in accuracy rate after a minimal delay (e.g. LaRooy et al. 2013; Schreiber Compo et al. 2011). However, given that recall is least impaired when tested straight away rather than with a delay (see Schreiber-Compo et al. 2017), future work should include both immediate and delayed recall measures to test whether intoxication acts at the encoding or the retrieval stage.

In line with previous evidence, whilst the recall of intoxicated participants was no less accurate than that of their sober counterparts, *in general*, intoxicated participants recalled fewer details overall (see Hagsand et al. 2013; Flowe et al. 2017). Moreover, for the cued recall questions, there was no effect of alcohol consumption on completeness. This concurs with a recent meta-analysis that showed an alcohol-related effect on completeness under free recall but not cued recall conditions (Jores et al. 2019). Whether the alcohol-related decrease in recall completeness is due to poor memory for the original event or due to changes in one’s metacognitive beliefs about their memory performance is unclear. Our confidence data suggests that alcohol does have an impact on participants’ metacognition. Although no less accurate in their free recall accounts, participants who were intoxicated reported significantly lower confidence than their sober counterparts. Again, this finding is in line with work showing a trend of underconfidence in intoxicated participants despite intoxication having no effect on recall accuracy (Crossland et al. 2016; Harvey et al. 2013). According to the source monitoring framework (Johnson and Raye 1981), individuals use perceptual, contextual and affective cues and cognitive operations that are present when memories are formed to determine the accurate source of that memory (Johnson et al. 1993). We showed that whilst alcohol produced underconfidence in these reports accurate source monitoring was not affected. Given the current sparsity of research examining the impact of alcohol on metacognition and the diversity of tasks used to measure it, more research is needed to disentangle the effects of alcohol on metacognitive regulation.

## Limitations

One limitation of the present study is the lack of mixed intoxication dyads. Studies have shown that sober participants are less likely to report contagion items proposed by a perceived intoxicated confederate (Thorley and Christiansen 2018). By including mixed dyads, the examination of how the intoxication of one’s partner may influence memory conformity could be investigated. In addition, asking participants to indicate how trustworthy or credible they found their discussion partner to be would be useful in helping to explain why intoxicated participants were not more susceptible to reporting misinformation from their co-mock witness. Future research would also benefit from the inclusion of a placebo (participants expect alcohol but receive no alcohol) and a reversed placebo group (participants do not expect alcohol but receive alcohol). Utilising a fully balanced placebo design, like Flowe et al. (2017) and Gawrylowicz et al. (2018) did, would allow to disentangle pharmacological effects of alcohol- and expectancy-related effects on memory conformity and recall performance.

A further limitation of the current study is that we did not probe participants for suspicion regarding the two different video versions. None of our participants expressed suspicion during the experiment and all acted genuinely surprised during the debrief when they were told that each dyad partner watched a different video version. Future research should probe participants for suspicion as it could yield important information.

Finally, laboratory studies typically reach BAC concentrations of < .08%. The mean BAC reached in the present study was 0.06%, which can be considered a low to moderate dose of alcohol. Low to moderate intoxication levels have been shown to cause changes in cognition in other domains such as driving hazard perception (West et al. 1993) and sustained attention (Magrys and Olmstead 2014). However, it could be argued that it is a lower dose of alcohol intoxication than what would be seen in real-world drinking scenarios. As such, future research should investigate the effect of memory conformity at higher intoxication doses (> .10%).

## Applied implications and conclusions

Contrary to the perceptions of laypeople (Benton et al. 2006; Wenger and Bornstein 2006) and professionals working within the criminal justice system (Crossland et al. 2018; Evans et al. 2009; Kassin et al. 2001), our findings suggest that mild to moderate alcohol intoxication does not make individuals more susceptible to incorporate misleading information obtained from a co-witness. Our work also shows that alcohol does impact recall completeness but not accuracy, so mild to moderately intoxicated witnesses may be regarded as a reliable source of information, even if questioned in an intoxicated state (when the delay is minimal). There is the potential for all witnesses, sober and intoxicated alike, who discuss a crime with a co-witness, to report information that they did not see but have just heard about from the other witness. Alcohol does not seem to exacerbate the memory conformity effect. It may therefore be important, regardless of whether witnesses are intoxicated or sober, to minimise co-witness discussion to prevent conformity effects on recall. Finally, police, judges and jurors should be made aware that intoxicated witnesses might report fewer details and might be less confident in their accounts. However, this does not necessarily mean that their accounts are less accurate or that they are less likely to accurately determine the source of the recalled information. Our study adds to the accumulating body of research demonstrating that an intoxicated witness is not necessarily an unreliable witness.
